# Head-to-Head Comparison between ^18^F-FES PET/CT and ^18^F-FDG PET/CT in Oestrogen Receptor-Positive Breast Cancer: A Systematic Review and Meta-Analysis

**DOI:** 10.3390/jcm11071919

**Published:** 2022-03-30

**Authors:** Arnoldo Piccardo, Francesco Fiz, Giorgio Treglia, Gianluca Bottoni, Pierpaolo Trimboli

**Affiliations:** 1 Nuclear Medicine Department, Ente Ospedaliero “Ospedali Galliera”, 16128 Genoa, Italy; francesco.fiz@galliera.it (F.F.); gianluca.bottoni@galliera.it (G.B.); 2 Faculty of Biomedical Sciences, University della Svizzera Italiana (USI), 6900 Lugano, Switzerland; giorgio.treglia@usi.ch (G.T.); pierpaolo.trimboli@usi.ch (P.T.); 3 Faculty of Biology and Medicine, University of Lausanne, 1015 Lausanne, Switzerland; 4 Academic Education, Research and Innovation Area, General Directorate, Ente Ospedaliero Cantonale, 6500 Bellinzona, Switzerland; 5 Clinic of Endocrinology and Diabetology, Lugano and Mendrisio Regional Hospital, Ente Ospedaliero Cantonale, 6500 Bellinzona, Switzerland

**Keywords:** PET/CT, FDG, FES, breast cancer, oestrogen receptor, diagnosis, nuclear medicine

## Abstract

^18^F-FDG PET/CT is a powerful diagnostic tool in breast cancer (BC). However, it might have a reduced sensitivity in differentiated, oestrogen receptor-positive (ER+) BC. In this setting, specific molecular imaging with fluorine-oestradiol (^18^F-FES) PET/CT could help in overcoming these limitations; however, the literature on the diagnostic accuracy of this method is limited. We therefore planned this systematic review and meta-analysis to compare ^18^F-FDG and ^18^F-FES PET/CT in ER+ BC patients. We performed a literature search to identify all studies performing a head-to-head comparison between the two methods; we excluded review articles, preclinical studies, case reports and small case series. Finally, seven studies were identified (overall: 171 patients; range: 7–49 patients). A patients-based analysis (PBA) showed that ^18^F-FDG and ^18^F-FES PET/CT had a similar high pooled sensitivity (97% and 94%, respectively) at the lesion-based analysis (LBA), ^18^F-FES performed slightly better than ^18^F-FDG (pooled sensitivity: 95% vs. 85%, respectively). Moreover, when we considered only the studies dealing with the restaging setting (n = 3), this difference in sensitivity was even more marked (98% vs. 81%, respectively). In conclusion, both tracers feature an excellent sensitivity in ER+ BC; however, ^18^F-FES PET/CT could be preferred in the restaging setting.

## 1. Introduction

^18^F-FDG PET/CT is a very useful imaging tool in staging breast cancer (BC) patients with locally advanced disease [1,2,3] or in restaging patients with biochemical relapse without evidence of structural recurrence on conventional imaging procedures [4,5]. However, the ^18^F-FDG avidity of BC is associated with histopathological features and can thus show relevant variations [6,7]. In particular, patients with less differentiated, triple-negative BC often present with ^18^F-FDG avid metastases and, in this setting, ^18^F-FDG PET/CT can provide relevant additional information when compared with morphological imaging modalities or with surgical staging [8]. Conversely, well-differentiated and oestrogen receptor-positive (ER+) BCs often present metastases with relatively low glucose consumption and thus inconstant positivity on ^18^F-FDG PET/CT. Therefore, in this particular field of patients, more specific PET/CT agents, such as receptor tracers, still represent an unmet diagnostic need.

Fluorine-oestradiol ^18^F-FES PET/CT has been introduced as an effective imaging procedure in patients with metastatic BC and previous histological confirmation of ER+ status on the primary tumour, to assess whether ERs are also expressed in loco-regional or distant metastases [9]. Indeed, ^18^F-FES PET/CT provides excellent whole-body information about heterogeneity in ER expression in BC metastases, thus allowing to predict the response to endocrine therapy [10]. However, little can be said about the diagnostic role of this receptor-specific tracer and, although some studies proved its low sensitivity in detecting BC liver metastases [11,12], others showed its relevant diagnostic impact on BC bone metastases [11,13]. Overall, it is not clear yet whether ^18^F-FES PET/CT could be proposed as a core imaging procedure in ER+ BC patients with suspected distant metastases and whether it could be preferred to ^18^F-FDG PET/CT, at least in some specific instances.

To clarify this diagnostic issue, we performed a systematic search of the literature to identify original studies reporting a diagnostic head-to-head comparison of ^18^F-FES PET/CT and ^18^F-FDG PET/CT in detecting ER+ BC metastases. We also carried out a meta-analysis of the available data using sensitivity as a diagnostic outcome measure.

## 2. Materials and Methods

The systematic review was conducted according with the PRISMA statement [14].

### 2.1. Search Strategy

Two authors (A.P. and G.B.) searched the available literature independently. The search and selection process consisted of four separate steps.

In the first step, so-called “sentinel” studies were identified in PubMed by entering various combinations of the following keywords: ^18^F-FES, ^18^F-FDG, PET/CT, breast cancer and oestrogen receptor. In the second step, the results were used to identify specific MeSH terms in PubMed. In the third step, PubMed, CENTRAL, Scopus, Web of Science and the web were searched using the selected MeSH terms. In the final step, we evaluated the studies that compared the sensitivity of ^18^F-FES PET/CT and ^18^F-FDG PET/CT in identifying disease localization in patients affected by ER+ BC. All initially eligible articles were screened, and those reporting a head-to-head comparison of ^18^F-FES PET/CT and ^18^F-FDG PET/CT in BC patients were included. Review articles, studies based on preclinical data, phantom studies, case reports, small case series and studies with overlapping data were excluded.

The references of the included studies were searched to identify further potential matches. The search process was concluded on 21 December 2021.

### 2.2. Data Extraction

The two authors (A.P. and G.B.) extracted independently:

1. General characteristics of the studies (authors, year of publication, country, study design, population).
2. Technical parameters (mode of acquisition, fasting before ^18^F-FDG injection and premedication, mean injected activity, uptake time, interval elapsed between the two imaging procedures, PET/CT scan field of view, PET/CT image analysis and use of reference standard).
3. Sensitivity of the two imaging procedures: this parameter was computed as a patient-based analysis (PBA) and a lesion-based analysis (LBA).
4. Standard of reference (SOR).

In the evaluation phase, full-text articles and their supplementary materials were included; in case of missing data, the responsible corresponding authors were contacted via e-mail. The extracted data were cross-checked and any discrepancy was discussed through a consensus meeting.

### 2.3. Study Quality Assessment

The risk of bias of included studies was assessed by one author (F.F.) using the QUADAS-2 method [15]. Briefly, for each study, an evaluation of the seven QUADAS-2 items was performed and each point was scored as having high, low or unclear risk of bias. High and unclear risk of bias were assigned 1 and 0.5 points, respectively; studies that totalled four or more points were excluded from the meta-analysis.

### 2.4. Statistical Analysis

A proportion meta-analysis was carried out using a random- effects statistical model. Pooled data are presented with their 95% confidence interval (95% CI) values. Heterogeneity across studies was assessed with the I-square statistic (I^2^): a value of 50% or higher was considered as a high heterogeneity. Publication bias was evaluated with Egger’s test [16].

The multidisciplinary follow-up was used as SOR to be able to compute the sensitivity of both image modalities (PBA/LBA). The OpenMeta[Analyst] statistical software (CEBM, Providence, RI, USA) was used for the statistical analyses.

## 3. Results

### 3.1. Literature Search Outcome

**Figure 1:**
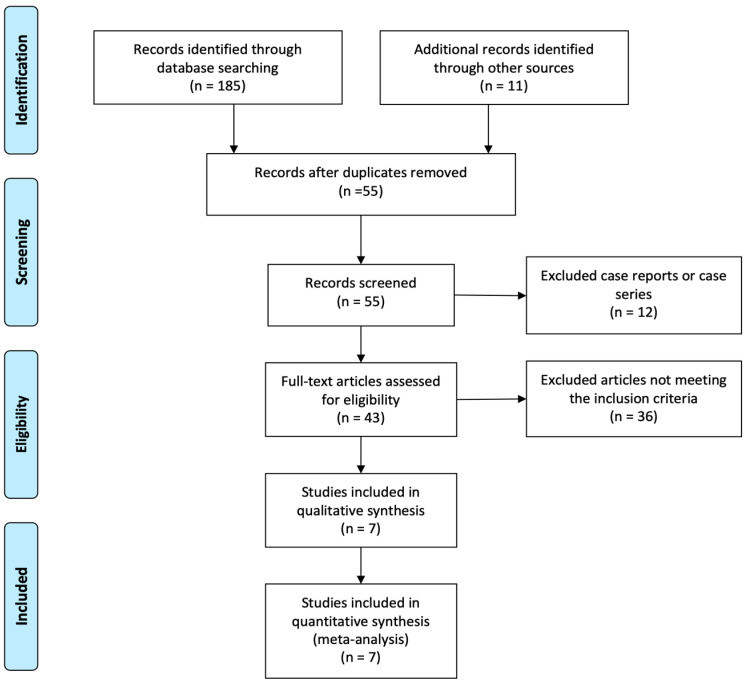
PRISMA flowchart, depicting the studies selection criteria.

A total of 55 records were initially identified after duplicate removal, and their titles and abstracts were assessed; 12 articles had to be excluded since they reported single cases or small cases series. Out of the remaining 43 records, 36 were excluded because they did not meet the set inclusion criteria. Therefore, seven articles were finally selected [12,17,18,19,20,21,22], including 171 ER+ BC patients (Figure 1).

### 3.2. Qualitative Analysis (Systematic Review)

The seven articles included in the systematic review had been published between 2013 and 2021. All of them had a retrospective design. Three studies were carried out in China, while India, Republic of Korea, USA and Italy, contributed one study each. The characteristics of the studies and their patients’ populations are summarized in Table 1.

#### 3.2.1. Technical Aspects

The imaging modality consisted of PET/CT with low-dose computed tomography settings in all cases. Information on fasting before ^18^F-FDG (4–6 h) injection were available in all articles. On the other hand, fasting was not required before ^18^F-FES injection.

The injected radiotracer activity ranged from 185 to 370 MBq for ^18^F-FDG PET/CT and from 111 to 222 for ^18^F-FES PET/CT. The time interval between radiotracer injection and PET/CT image acquisition was similar across the studies, being 60 min for both tracers in the majority of cases but one [19] in which ^18^F-FES PET/CT acquisition started 80–100 min after the tracer injection.

In all studies, PET image analysis was performed by a combination of qualitative (visual) and semi-quantitative analysis through the calculation of the maximum standardized uptake values (SUV_max_).

On visual analysis, ^18^F-FDG focal uptake greater than the surrounding normal tissue that could not be explained by physiological activity was considered as positive in five studies [12,18,20,22,23]. In the remaining two studies, the criteria to classify ^18^F-FDG PET findings as positive were not clearly specified [17,21]. When ^18^F-FES PET/CT was considered, in three studies a SUV_max_ cut-off was introduced to interpret as positive each focal tracer uptake [18,20,22]. On the other hand, a visual interpretation (i.e., uptake higher than surrounding background) was used in another study [12], and in a further three analyses, the criteria were not reported [17,19,21]. All technical aspects are summarized in Table 2.

#### 3.2.2. Diagnostic Performance

The seven articles selected for the systematic review were published between 2013 and 2021 and included populations consisting of 7 to 49 patients affected by ER+ BC (Table 1). Table 3 details the rate of positive cases at the PBA and LBA.

Overall, ^18^F-FDG PET/CT and ^18^F-FES PET/CT showed very high sensitivity in detecting sites of disease in ER+ BC patients. Indeed, in two studies, ^18^F-FDG PET/CT identified more patients with BC lesions/metastases than ^18^F-FES PET/CT [19,22]. Conversely, in one study, ^18^F-FES PET/CT showed more BC lesions than ^18^F-FDG PET/CT [21]. In the other four studies [12,17,18,20], both diagnostic procedures identified at least one BC lesion in the 75 patients analysed. Overall, no significant differences between the two methods were reported.

When the ability for detecting each single lesion/metastasis was considered (i.e., LBA), ^18^F-FES PET/CT identified more BC lesions in three studies [18,21,22] and ^18^F-FDG PET/CT identified more BC lesions in another three studies [12,19,20]. In one study, including only patients with primary BC, both modalities identified the same number of lesions [17]. As the main finding, we can point out that the studies reporting a higher number of ^18^F-FDG-positive lesions included patients often affected by ductal carcinoma (i.e., 96%) [19] among which were also included those with ER+ HER2 + BC (11%) [19] and those with liver metastases (>10%) [12]. In addition, these patients were studied at a time of suspicious relapse when heterogeneity, due to the comparison of metastatic ER- clones, is more frequent [12,20]. On the other hand, studies showing a higher number of ^18^F-FES-positive lesions often analysed patients affected by lobular BC [21] or patients with a high prevalence of bone metastases [22]. Moreover, patients were predominantly affected by ER+ HER- BC [18,22] and were often studied at the time of their first staging [18].

When the impact on clinical decision making was considered, only one study [18] reported that ^18^F-FES PET/CT was able to change therapeutic strategies in 5 out of the 19 (26.5%) patients analysed at the time of first staging. Indeed, this diagnostic procedure was able to properly downstage two patients and upstage three when compared with ^18^F-FDG PET/CT.

#### 3.2.3. Quality Assessment of the Studies

The risk of bias was assessed according to seven items, which are listed in Table 4. The overall bias score ranged from none to 2.5; therefore, no study had to be excluded because of high bias risk. The most frequent sources of possible bias were the “study test” and “reference standard categories” since, in some studies, it was unclear whether a blinded evaluation of the two methods had been performed. In particular, in the study by Ulaner et al. [21], the same reader assessed both examinations. In opposition to this, risks regarding the feasibility were almost never detected.

### 3.3. Quantitative Analysis (Meta-Analysis)

**Figure 2:**
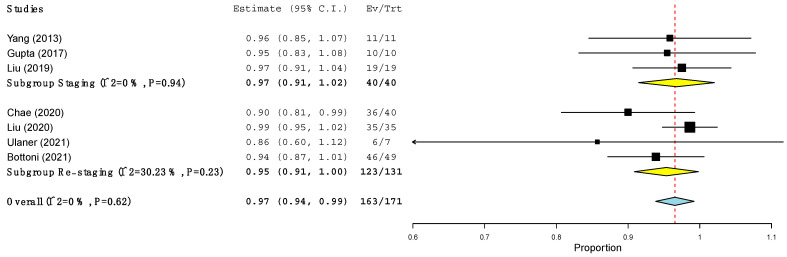
Sensitivity of ^18^F-FDG PET/CT at the level of patients across studies.

**Figure 3:**
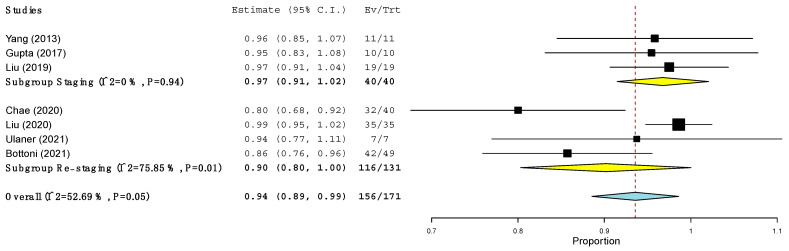
Sensitivity of ^18^F-FES PET/CT at the level of patients across studies.

**Figure 4:**
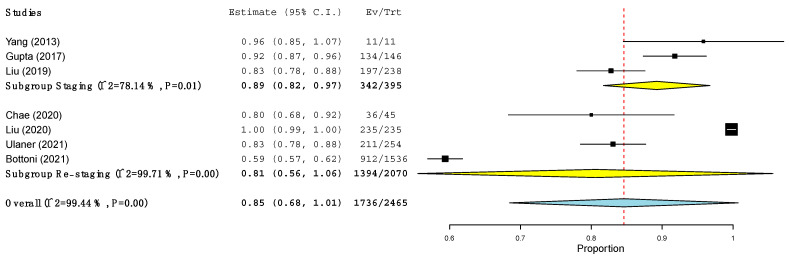
Sensitivity of ^18^F-FDG PET/CT at the level of the lesions across studies.

**Figure 5:**
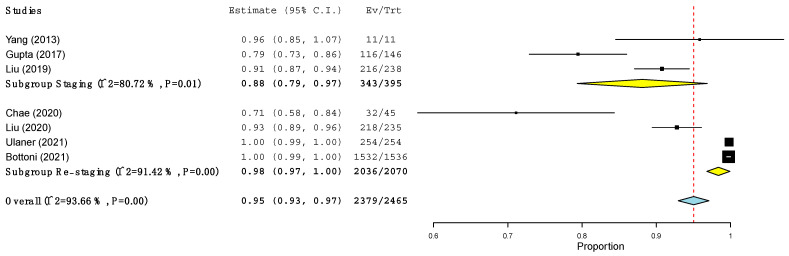
Sensitivity of ^18^F-FES PET/CT at the level of the lesions across studies.

The pooled sensitivity of ^18^F-FES PET/CT and ^18^F-FDG PET/CT in terms of PBA and LBA was computed (Table 5 and Figure 2, Figure 3, Figure 4 and Figure 5) based on the available data (see Table 3). Regarding PBA, the pooled sensitivity of ^18^F-FDG PET/CT and ^18^F-FES PET/CT was 97% and 94%, respectively, with overlapping 95% confidence intervals. In the LBA, however, we observed a wider difference between the pooled sensitivity of the two methods (95% for ^18^F-FES vs. 85% for ^18^F-FDG). Although the 95% confidence intervals of the two methods were overlapping, the values of ^18^F-FES PET/CT were consistently at the higher end of the spectrum (93–97%), while those of ^18^F-FDG PET/CT showed a much larger spread (68–100%). In all the analyses, high heterogeneity was found (Table 5).

On the basis of the above results, the heterogeneity of PBA and LBA was explored by using the following covariates: timing of the studies (i.e., PET/CT assessment on staging and on time of relapse), their sample sizes, the prevalence of ductal or lobular BC, and the prevalence of bone and liver metastases.

However, these last variables could not be tested, given incomplete and inconsistent data, and only the variable “timing of the studies” was explored. As illustrated in Figure 2 and Figure 3, the heterogeneity of the ^18^F-FES PET/CT and ^18^F-FDG PET/CT results was no longer present in the PBA when we considered these two groups separately. In fact, in the staging scenario, the sensitivity in the PBA of both the ^18^F-FES PET/CT and ^18^F-FDG PET/CT was 97% (Figure 2 and Figure 3). Conversely, at the time of restaging, the patient-based sensitivity of ^18^F-FES PET/CT and ^18^F-FDG PET/CT was 90% and 95% respectively (Figure 2 and Figure 3).

When LBA was conducted, the sensitivity of ^18^F-FES PET/CT and ^18^F-FDG PET/CT at the time of first staging was 88% and 89% respectively, while at the time of restaging it was 81% and 98%, respectively (Figure 4 and Figure 5). Interestingly, the sensitivity of ^18^F-FES PET/CT at the time of restaging was significantly higher than that of the same procedure at the time of staging (Figure 4).

## 4. Discussion

In this systematic review and meta-analysis, we aimed to clarify the diagnostic role of ^18^F-FES PET/CT in BC patients compared to ^18^F-FDG PET/CT. In fact, ^18^F-FES PET/CT has gained significant attraction as a non-invasive means to predict the effectiveness of hormone blockade. However, its potential in the mere diagnostic evaluation is still debated [1,8,21]. In this study, we gathered all the available evidence of its sensitivity in ER+ BC patients when compared with the more commonly used ^18^F-FDG PET/CT.

Our qualitative and quantitative assessment in this particular BC subpopulation showed that there are no significant differences in terms of sensitivity between the two PET tracers at the PBA. Indeed, both molecular imaging modalities proved able to detect patients affected by ER+ BC with similarly high sensitivity. The lack of significant difference between these two modalities can, however, be explained by the selection of patients included in the analysis. These patients were evaluated at the time of first staging for an already known primary tumour or who underwent PET at the time of relapse to evaluate the extension or the ER expression of the metastatic disease. This population is indeed characterized by a very high prevalence of a true positive BC lesion; the probability that at least one these lesions was detected by one of the two molecular imaging procedures was very high. The small diagnostic advantage of the ^18^F-FDG PET/CT over ^18^F-FES PET/CT (97% vs. 94%) reported in our analysis seems related to the variability in terms of the clinical characteristics of the patients. Indeed, the high prevalence of metastatic heterogeneity, often present at the time of restaging, can be associated to false negative ^18^F-FES PET/CT results. When the diagnostic performances of these two imaging procedures at the time of recurrence was explored by means of a PBA, the sensitivity of ^18^F-FDG PET/CT and that of ^18^F-FES PET/CT was 95% and 90%, respectively.

Conversely, when we investigated the diagnostic sensitivity of these diagnostic tools by means of an LBA, we found that the sensitivity of ^18^F-FDG PET/CT and that of ^18^F-FES PET/CT was 85% and 95%, respectively. Indeed, when the analysis was focused on the time of disease relapse, ^18^F-FES PET/CT was more sensitive than ^18^F-FDG PET/CT (98% vs. 81%) with a trend towards significance (95% CI: 97–100% and 56–100%, respectively).

Overall, the use ^18^F-FDG PET/CT as a first-line examination appears to be the best strategy to stage and restage the ER+ BC subjects, being able to identify the highest number of true positive patients. However, if this approach is applied to a population of ER+ BC with low metastatic heterogeneity (i.e., lobular breast cancer, or ductal breast cancer with a high percentage of ER positive and HER2 negative cells) it could underestimate the actual disease burden since such a clone could show low glucose avidity. Indeed, although ^18^F-FES PET/CT has low sensitivity in detecting liver metastases (given the intense background tracer uptake in this organ), it has a very high sensitivity in disclosing peripheral lesions in other organs or tissues, such as bone lesions, which represent the most frequent sites of disease in ER+ BC [22]. In addition, as reported by Gupta et al. [12], one of the diagnostic advantages of this receptor PET tracer is its higher specificity in characterizing cervical, axillary and mediastinal lymph nodes, which may often present unspecific uptake at ^18^F-FDG PET/CT and thus be misinterpreted on ^18^F-FDG PET/CT.

Although our systematic review and meta-analysis reported interesting and useful results to understand the weaknesses and strengths of the two imaging procedures, some limitations should also be borne in mind. First, only seven studies, investigating relatively small patient populations, were fit for inclusions in this meta-analysis. In addition, all these seven studies showed a retrospective design, and three out of seven included a low number of ER+ BC patients. However, the selection criteria limited us to studies that tested both ^18^F-FDG PET/CT and ^18^F-FES PET/CT within a restricted time frame. Second, a computation of specificity was not possible since none of the studies reported the true negative rate. Third, the absence of histological confirmation of suspected distant metastases detected by both modalities is an important bias that could have affected some of the studies included in this analysis [12,20,21,22], and we cannot exclude the possibility that some of the metastases detected by PET tracers may have been false-positive findings. However, given the elevated prevalence of patients with disseminated disease, the likelihood of any given finding to be a false negative was relatively low. Moreover, ethical and practical reasons prevented the execution of a biopsy evaluation of each single lesion. In addition, in at least 3 out of 7 studies, a multidisciplinary follow-up (consisting of clinical and imaging evaluation) was available. Fourth, only two out of the seven studies reported information regarding the menopausal status of the patients. Indeed, these data could be of value to correctly estimate the real sensitivity of ^18^F-FES PET/CT that could theoretically be affected by competitive binding of high oestradiol concentration. However, the exact endogenous oestrogen concentration that can have a measurable effect on tumour ^1^⁸F-FES uptake remains hitherto unexplored [9]. Statistically significant heterogeneity of the ^18^F-FES PET/CT pooled heterogeneity was found across studies. Regrettably, the sparse data available in the studies did not allow exploration of the heterogeneity with the exception of the variable “timing of the studies”.

Finally, at this time, data about the cost/effectiveness of ^18^F-FES PET/CT in comparison with that of ^18^F-FDG PET/CT do not exist. However, it is conceivable that, when the overall expense is considered, the additional information provided by FES might help in optimizing the cost by guiding the choice of the most appropriate therapeutic protocol.

## 5. Conclusions

The present data show that both ^18^F-FDG PET/CT and ^18^F-FES PET/CT represent accurate imaging procedures in patients with ER+ BC, providing comparable results in terms of sensitivity. However, in the field of lesion detection, we observed a non-negligible difference in favour of ^18^F-FES PET/CT. Thus, the use of ^18^F-FES PET/CT as a first-line molecular imaging procedure might be considered in lobular breast cancer or ductal breast cancer with a high percentage of ER and HER2 negative. However, larger, multicentre and prospective studies are required to confirm these preliminary indications.

## Figures and Tables

**Table 1:** Study and patient characteristics.

Authors	Year	Country	Study Design	Patients	ER+ BC *	Ductal/Lobular	HER2+	Pre/Post-Menopause	Staging/Restaging	Liver Metastases Analysed	SOR
Yang et al. [17]	2013	China	R	18	11	11/0	10	NR	11/0	No	Histopathology *
Gupta et al. [12]	2017	India	R	10	10	NR	N.R.	NR	5/5	Yes	N.R. *
Liu et al. [18]	2019	China	R	19	19	NR	N.R.	NR	19/0	No	Histopathology *
Chae et al. [19]	2020	Korea	R	46	40	38/2	5	13/33	0/40	No	Histopathology *
Liu et al. [20]	2020	China	R	35	35	29/4 **	0	7/28	0/35	No	Multidisciplinary ***
Ulaner et al. [21]	2021	USA	R	7	7	0/7	0	NR	0/7	Yes	Multidisciplinary ***
Bottoni et al. [22]	2021	Italy	R	49	40	N.R.	0	NR	0/49	No	Multidisciplinary ***

* Legend: R = retrospective; NR = not reported; SOR = standard of reference. ** Two patients were affected by mucinous and tubular BC, respectively. *** Clinical and imaging-based follow-up.

**Table 2:** Technical aspects of PET imaging in the included studies.

Authors	Hybrid Imaging Modality	PET/CT Tomograph	Patient Preparation	Mean Radiotracer Injected Activity	Time Interval between Radiotracer Injection and Image Acquisition	Timeframe between the Two PET/CT	Image Analysis	^18^F-FES PET/CT Interpreted as Positive When
Yang et al. (2013) [17]	PET/CT with low-dose CT	Siemens Biograph 16 HR	For ^18^F-FDG fasting (4 h)	^18^F-FDG: 7.4 MBq/Kg ^18^F-FES:222 MBq	60 min for both tracers	Up to 7 days	Visual and semi-quantitative (SUV_max_)	NR
Gupta et al. (2017) [12]	PET/CT with low-dose CT	Siemens Biograph TruePoint 40	For ^18^F-FDG fasting (4 h)	^18^F-FDG: 4–5 MBq/Kg ^18^F-FES: 200 MBq	60 min for both tracers	Up to 7 days	Visual and semi-quantitative (SUV_max_)	^18^F-FES uptake higher than surrounding background
Liu et al. (2019) [18]	PET/CT with low-dose CT	Siemens Biograph 16 HR	For ^18^F-FDG fasting (6 h)	^18^F-FDG: 7.4 MBq/Kg ^18^F-FES: 222 MBq	60 min for both tracers	Up to 7 days	Visual and semi-quantitative (SUV_max_)	^18^F-FES uptake higher than surrounding background (SUV_max_ > 1.8)
Chae et al.(2020) [19]	PET/CT with low-dose CT	Siemens Biograph Sensation 16 or Biograph TruePoint 40; or GE Discovery 690, 690 Elite, or 710	For ^18^F-FDG fasting	^18^F-FDG: 5.2–7.4 MBq/Kg ^18^F-FES:111–222 MBq	80–100 min for ^18^F-FES 50–70 min for ^18^F-FDG	Median 10 days	Visual and semi-quantitative (SUV_max_)	NR
Liu et al. (2020) [20]	PET/CT with low-dose CT	Siemens Biograph 16 HR or mCT Flow	For ^18^F-FDG fasting (6 h)	^18^F-FDG: 3.7–7.4 MBq/Kg ^18^F-FES: 222 MBq	60 min for both tracers	Up to 28 days	Visual and semi-quantitative (SUV_max_)	^18^F-FES uptake higher than surrounding background (SUV_max_ > 1.8)
Ulaner et al. (2021) [21]	PET/CT with low-dose CT	NR	For ^18^F-FDG fasting (6h)	^18^F-FDG: 444–555 MBq ^18^F-FES: 185 MBq	60 min for both tracers	Up to 35 days	Visual and semi-quantitative (SUV_max_)	NR
Bottoni et al. (2021) [22]	PET/CT with low-dose CT	GE Discovery ST, Discovery LS or Siemens Biograph mCT Flow	For ^18^F-FDG fasting (4–6 h)	^18^F-FDG: according to the patient’s body weight (Boellaard R. et al. EJNMMI 2014) ^18^F-FES: 200 MBq	60 min for both tracers	Up to 10 days	Visual and semi-quantitative (SUV_max_)	^18^F-FES uptake higher than surrounding background (SUV_max_ > 2)

Legend: CT = computed tomography; MBq = megabecquerel; NR = not reported; PET/CT = positron emission tomography; SUV_max_ = maximal standardized uptake value.

**Table 3:** Data available in the seven studies included in the present systematic review.

First Author [ref]	Patients	^18^F-FES PET/CT	^18^F-FDG PET/CT	Lesions	^18^F-FES PET/CT	^18^F-FDG PET/CT
	n (tot)	+ve	+ve	n (tot)	+ve	+ve
Yang et al. (2013) [17]	11	11	11	11	11	11
Gupta et al. (2017) [12]	10	10	10	146	116	134
Liu et al. (2019) [18]	19	19	19	238	216	197
Chae et al.(2020) [19]	40	32	36	45	32	36
Liu et al. (2020) [20]	35	35	35	235	218	235
Ulaner et al. (2021) [21]	7	7	6	254	254	111
Bottoni et al. (2021) [22]	49	42	46	1536	1532	912

Legend. +ve: positive.

**Table 4:** Quality assessment of the studies and risk of bias in each study considered.

		Risk of Bias				Feasibility		
First Author	Year	Patient Selection	Study Test	Reference Standard	Timing	Patient Selection	Study Test	Reference Standard
Yang et al. [17]	2013	L	L	L	L	L	L	L
Gupta et al. [12]	2017	H	U	L	L	H	L	L
Liu et al. [18]	2019	L	U	U	L	L	L	L
Chae et al. [19]	2020	L	L	L	U	L	L	L
Liu et al. [20]	2020	L	U	U	L	L	L	L
Ulaner et al. [21]	2021	L	H	H	L	L	L	L
Bottoni et al. [22]	2021	L	U	U	L	L	L	L

Legend: H = high, L = low, U = unclear.

**Table 5:** Pooled sensitivity for PBA and LBA of ^18^F-FES PET/CT and ^18^F-FDG PET/CT.

	PBA			LBA		
	Sensitivity (95% CI)	I^2^	Egger’s Test (*p*)	Sensitivity (95% CI)	I^2^ (%)	Egger’s Test (*p*)
^18^F-FES PET/CT	94% (89–99)	52.7%	*p* = 0.048	95% (93–97)	93.66%	*p* < 0.01
^18^F-FDG PET/CT	97% (94–99)	0%	*p* = 0.62	85% (68–100)	99.44%	*p* < 0.001

Legend: PBA, patient-based analysis; LBA, lesion-based analysis.

## Data Availability

Not applicable.
